# Non-cell-autonomous effects yield lower clonal diversity in expanding tumors

**DOI:** 10.1038/s41598-017-11562-w

**Published:** 2017-09-11

**Authors:** Tazzio Tissot, Frédéric Thomas, Benjamin Roche

**Affiliations:** 1 CREEC/MIVEGEC, UMR IRD/CNRS/UM 5290, 911 Avenue Agropolis, BP 64501, 34394 Montpellier, Cedex 5 France; 2 Unité mixte internationale de Modélisation Mathématique et Informatique des Systèmes Complexes. (UMI IRD/UPMC UMMISCO), 32 Avenue Henri Varagnat, 93143 Bondy Cedex, France

## Abstract

Recent cancer research has investigated the possibility that non-cell-autonomous (NCA) driving tumor growth can support clonal diversity (CD). Indeed, mutations can affect the phenotypes not only of their carriers (“cell-autonomous”, CA effects), but also sometimes of other cells (NCA effects). However, models that have investigated this phenomenon have only considered a restricted number of clones. Here, we designed an individual-based model of tumor evolution, where clones grow and mutate to yield new clones, among which a given frequency have NCA effects on other clones’ growth. Unlike previously observed for smaller assemblages, most of our simulations yield lower CD with high frequency of mutations with NCA effects. Owing to NCA effects increasing competition in the tumor, clones being already dominant are more likely to stay dominant, and emergent clones not to thrive. These results may help personalized medicine to predict intratumor heterogeneity across different cancer types for which frequency of NCA effects could be quantified.

## Introduction

Patient biopsies reveal that most of solid tumors display a high diversity of cancer cell subtypes^1^. This intratumor heterogeneity (ITH) early emerges during carcinogenesis^2^ and is correlated with cancer progression^3^. It predicts both cancer aggressiveness^2, 4, 5^ and post-therapeutic recurrence risk^6, 7^. Two main mechanisms have emerged to explain the coexistence of numerous phenotypes within the same tumor^8, 9^. On the one hand, cell phenotypic plasticity, especially the existence of cancer stem cells, allows cells with a same genotype (i.e. a clone) to display different phenotypes, due to epigenetic differences or differences in cell signaling pathways^10, 11^. On the other hand, clonal evolution generates a range of genotypes across time, due to the accumulation of mutations in cancer cells’ genomes^12^.

Clonal diversity (CD) is the genetic component of phenotypic diversity displayed by tumors. It is yielded by the branching clonal evolution in progress in every tumor: i.e., the accumulation of mutations yields a range of clones that coexist in similar frequencies within the same micro-environment (ME)^13–15^. A part of these mutations can impact growth rates, and eventually the evolution of frequencies across time^16, 17^. Among them, beneficial mutations (known as *drivers*) usually speed up the growth kinetics of the carrier clones^18^, which may eventually lower CD^19^. If drivers are not rare in tumors and should negatively impact CD, some of them can nevertheless promote their carriers’ growth without increasing in frequency^20, 21^. Indeed, if mutations can impact their carriers’ growth through cell-autonomous effects (CA effects), they can also impact other noncarrier clones’ growth, through non-cell-autonomous effects (NCA effects^22, 23^).

The significance of NCA effects for CD is increasingly addressed in cancer research^23–25^. NCA effects of mutations were found to directly impact the growth of noncarrier clones by many different mechanisms (Supporting information, Table S1)^23^. This body of NCA effects is already taken into account in the design of patient-derived models of tumors to yield similar ITH *in vitro* as *in vivo* ^22^, but *in silico* models of CD evolution have very poorly addressed the impacts of NCA effects so far. Most of models studying ITH implicitly exclude NCA effects^17, 19, 26, 27^, while models involving NCA effects in tumor evolution do not measure the impacts on ITH^28, 29^. Though, in a data-fitted model, Marusyk *et al*. predicted a higher CD on the long run when a single clone could non-cell-autonomously promote the exponential growth of a limited number of other clones^21^. But how numerous different NCA effects impact CD on the long run in the tumor remains unclear.

In the absence of NCA effects, only neutral and beneficial mutations persist. The latter accelerate the emergence of new clones through divisions, which consequently supports CD on the long run. However, when clones carry mutations with NCA effects, they could also shape the growth kinetics of noncarrier clones. On the one hand, mutations with NCA effects could balance the growth kinetics of the diversity of preexisting clones in the tumor: beneficial mutations with NCA effects could allow slow growing clones (potentially carrying detrimental mutations) to persist, and detrimental mutations with NCA effects could mitigate the outgrowth of fast growing clones. This would allow the coexistence of clones at similar frequencies, and thus support CD. On the other hand, mutations with NCA effects could exaggerate the differences in growth kinetics already existing between clones: detrimental mutations with NCA effects could also limit the probability for emerging clones to reach high frequencies, and beneficial mutations with NCA effects could accelerate the growth kinetics of already fast growing clones. Without necessarily limitating clonal richness, this would exacerbate the differences between clonal frequencies and limit equitability, thus reducing CD^30^. The possible outcomes of CD when tumor growth is driven by NCA effects, are therefore challenging to forecast.

In order to test the impact of NCA effects on CD evolution, we follow the branching of clones interacting with each other through the NCA effects of their mutations. Since mathematical models of cell growth have tractability issues at simultaneously considering large numbers of clones, we choose the framework of individual-based models^31^. In our model, cells divide and die according to division and death rates determined by CA and NCA effects. Across divisions, cells can mutate and therefore produce new clones. In order to stay apart from spatial issues, we assume that the diffusion of NCA effects is instantaneous from one cell to another and does not depend on the density of cells in the tumor. This model allows quantifying CD (assumed to be represented by Simpson index) across time and divisions, depending on the frequency of mutations with NCA effects. Thus, in order to disentangle the link between NCA-driven growth kinetics and CD, we simulated tumors growing either during a fixed number of generations, or until they reached a fixed size, whereas CA and NCA effects had various impacts on clonal growth kinetics.

## Methods

**Figure 1:**
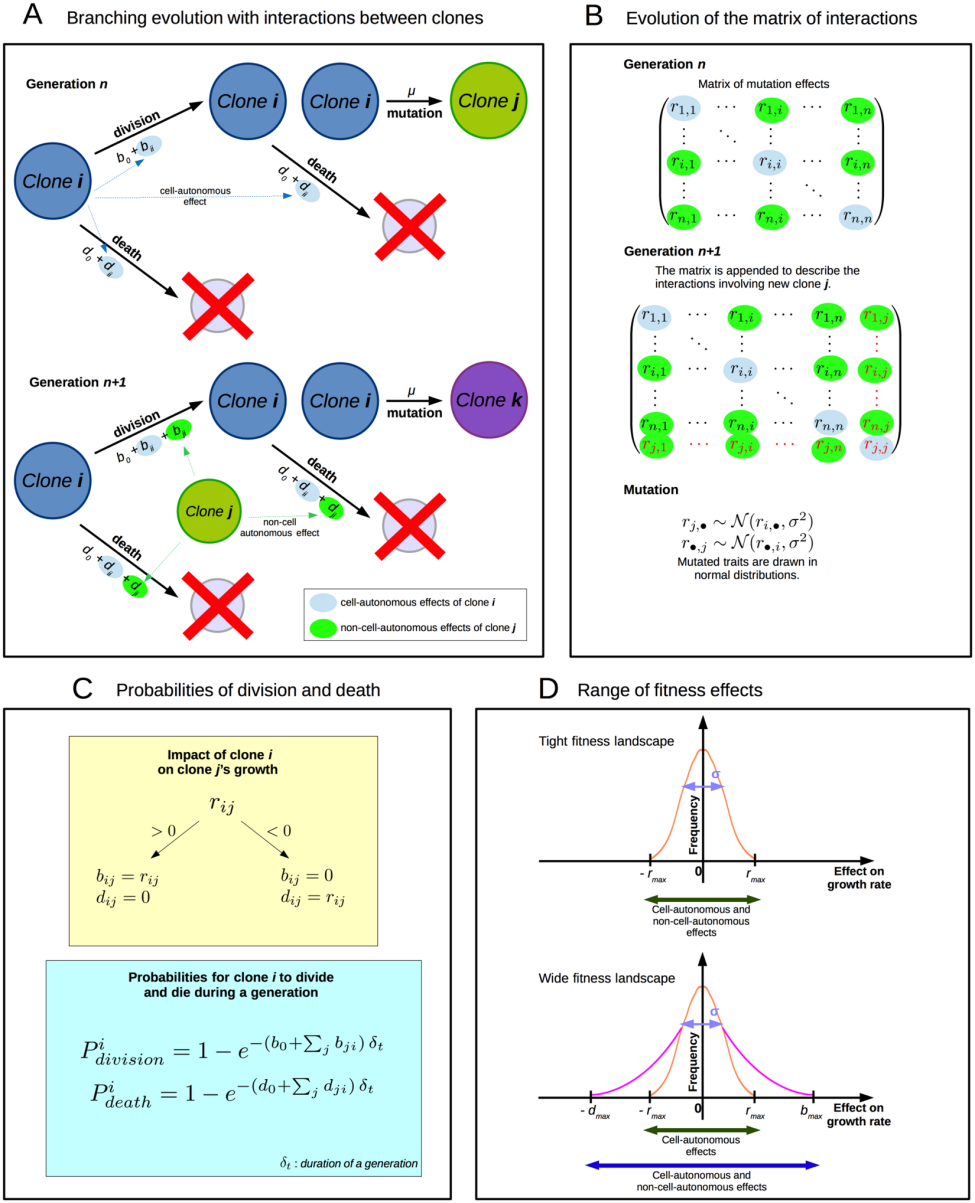
Individual-based model of clonal evolution with interactions. (**A**) Model architecture: division and death rates depend both on basal rates (*b*_0_ and *d*_0_) and on the effects of mutations on phenotype. Mutations affect clonal growth kinetics through a matrix of interactions, where *b*_*ji*_ and *d*_*ji*_ are the effects of clone *j* on clone *i*’s growth. (**B**) Evolution of the matrix of interactions: the matrix of interactions encompasses both cell-autonomous effects (*b*_*ii*_ and *d*_*ii*_) and non-cell-autonomous effects (*b*_*∙*,*i*_ and d_*∙*,*i*_). When new clones arise by mutation, the matrix is appended with new interaction coefficients *r*_*∙*,*∙*_ which are drawn in normal distributions centered on interaction coefficients of the resident clone. (**C**) Probabilities of division and death: interactions coefficients are distinguished on whether they benefit or harm the recipient clone. They are then added to basal division and death rates to produce effective division and death rates. These effective rates are then used as parameters for exponential distribution in which division and death events are drawn. (**D**) Range of fitness effects: in a tight fitness landscape, the maxima of division and death rates are set to the range of mutation effects distribution, *i*.*e*., cell-autonomous and non-cell-autonomous effects similarly impact the division and death rates. In a wide fitness landscape, the maxima of division and death rates are higher than the maxima of mutation effects distribution, *i*.*e*., non-cell-autonomous effects can have distinct impacts on clonal growth rates from cell-autonomous effects’.

The goal of our individual-based model is to implement the stochastic evolution of interacting, clonally-reproducing cells, allowing us to monitor CD for a range of conditions in mutations, tumor age and tumor size. The model is described in Text S1 and Fig. 1, and the main algorithm is detailed in Text S2.

### Model behavior

We start each simulation with a self-replicating population of ${N}_{{\rm{0}}}$ noncancerous cells. These cells divided and died at the exact same basal rate ${b}_{{\rm{0}}}={d}_{0}$, and thus their population size only experienced stochastic fluctuations, corresponding to physiological conditions of self-renewing stem cells in tissues. Across divisions, each cell had a probability *μ* to mutate and to produce a new clone, which we considered to be cancerous (see Fig. 1A). At each timestep, probabilities of division and death events were computed for each cell, before to be eventually applied, so that these events could be implemented in any order without influencing their respective outcomes:

1 $${P}_{division}^{i}={\rm{1}}-\exp (-({b}_{{\rm{0}}}+\sum _{j}{b}_{ij})\cdot {\delta }_{t})$$

2 $${P}_{death}^{i}={\rm{1}}-\exp (-({b}_{{\rm{0}}}+\sum _{j}{d}_{ij})\cdot {\delta }_{t})$$

for *i* being the identity of the clone. According to these equations, every new clone grew exponentially according to basal division and death rates, but mutations could also confer them growth advantages or losses that subsequently raised or lowered their population sizes. These mutation effects on division and death rates were drawn in a fitness landscape, and could be either CA (only affecting the clone carrying the mutation, *r*_*ii*_ for an impact of clone *i* on its own division and death rates) or NCA (possibly affecting other clones, *r*_*ij*_ for an impact of clone *i* on clone *j*’s division and death rates, see Fig. 1B–C). The values of mutation effects could be either positive or negative, and were add up to basal division and death rates according to their sign: positive mutations were add up to division rates, *i*.*e*., *b*_*ji*_ = *r*_*ji*_ if *r*_*ji*_ ≥ 0; and negative ones to death rates, *i*.*e*., *d*_*ji*_ = *r*_*ji*_ if *r*_*ji*_ ≤ 0. CA and NCA effects were considered as frequency- and density-independent, *i*.*e*., any clone could non-cell-autonomously impact others, provided it was composed of at least one cell. The values of mutation effects could be either positive or negative: effective division rates were then computed by adding all the positive mutation effects to the basal division rate, and effective death rates by adding all the negative mutation effects to the basal death rate. In order to keep an exponential growth of cancer cells^32^, division and death rates could not exceed maximal rates *b*_*max*_ = *d*_*max*_, and mutation effects could not produce fitness variations higher than *r*_*max*_ (Supporting information, Table S2 for values). Besides, cancerous cells were as likely as noncancerous cells to mutate and to produce new clones.

Most mutations do not produce large fitness variations during cancer^33^, even though most cancers have their genomes highly instable^34, 35^. Mutated traits were thus drawn in normal distributions centered on the resident traits, with standard deviation *σ* (see Fig. 1B). This ensured that new mutants were phenotypically close from the clones that they derived from, in order to let mutations accumulate in clones. Among mutation events, we enforced different frequencies of mutations with NCA effects, ranging from 0 to 50% of mutations. Besides, in order to distinguish the impacts of NCA effects on growth kinetics from those of CA effects, we performed simulations for two distinct fitness landscapes (see Fig. 1D). In the condition referred to as *tight fitness landscape*, the maxima of division and death rates were fixed to *r*_*max*_. In the condition referred to as *wide fitness landscape*, the maxima of division and death rates were fixed to *b*_*max*_ = *d*_*max*_.

We considered that the whole cell population formed a tumor as soon as the whole cell population (as a sum of all clone sizes) has grown enough to consider that cancerous clones are driven to cancer outgrowth (tumor formation is assumed to have been reached at $N={\alpha }_{t}\cdot {N}_{{\rm{0}}}$). We then recorded the emergence of new clones and the size of every clone at each generation. In order to determine the impact of NCA effects on CD, simulations could stop when they reached a final condition of fixed time or fixed size: (i) either when tumor evolution had been recorded during a given number of generations *t*_*max*_ = 300 generations; (ii) or when tumor had exceeded a given size *N*_*max*_ = 3.10^5^ cells. These two conditions allowed to track the evolution of CD during the very first steps of cancer progression, when diversity emerges^36^.

This framework is based on parameters which have rarely been addressed in empirical studies, and mostly in theoretical studies. We thus performed simulations on parameter values used in similar models (Supporting information, Table S2). Even though these values were not all biologically motivated, they allowed us to compare our results with other studies addressing NCA effects and CD. In order to test the sensitivity of CD and growth kinetics to other parameters, the parameter space was explored through a method of Latin Hypercube Sampling (LHS^37^) as implemented in function randomLHS of the statistical software R^38^. LHS was performed on uniform distributions (rather than normal distributions, in order to alleviate the uncertainty on parameter values) bounded in 20% variations of the values displayed in Table S2 (see “80% Value” and “120% Value”). In order to avoid outliers among simulations, we did not record simulations for which tumor size did not reach ${\alpha }_{t}\cdot {N}_{{\rm{0}}}$ cells within 10000 generations, or *N*_0_ cells within 1500 generations after tumor formation, nor those which dropped below *N*_0_ cells (from 1 to 10% of simulations, depending of the condition tested).

### Data analysis

For each condition, we performed 500 simulations reaching the expected final status. At the end of each simulation, we quantified clonal diversity with Simpson index. This measure, which was previously used in other models addressing of tumor heterogeneity^19, 26^, accounts for both clonal richness and equitability, while being bounded. For *p*_*i*_ being the frequency of clone *i* in the tumor, Simpson index was computed as:

3 $$H={\rm{1}}-\sum _{i}{p}_{i}^{{\rm{2}}}$$

We obtained similar patterns when measuring clonal diversity with Shannon index (Supporting information, Fig. S1).

We also recorded the number of generations (tumor age) necessary to reach *N*_*max*_ cells and the tumor size after *t*_*max*_ simulations for simulations during a given time. In order to have a common measure of growth kinetics for both types of simulations, mean tumor growth rate was then computed for each simulation as:

4 $$Tumor\,growth\,rate=\frac{Tumor\,size}{Tumor\,age}$$

These measures were not normally distributed (assessed by a Shapiro-Wilk test), and did not follow homoscedasticity (assessed by a Brown-Forsythe test). Instead of an ANOVA, we thus performed linear regressions on both Simpson index and tumor growth rate as functions of the frequency of mutations with NCA effects. We kept models with maximal R^2^.

In order to discriminate the impacts of NCA effects relatively to other parameters, we performed a sensitivity analysis for Simpson index and tumor growth rate. We screened for both linear and nonlinear relationships with these measures, so that rank-transformation of the data was necessary. We thus computed partial rank correlation coefficients (PRCC^37^) depending on minimal tumor size ${\alpha }_{t}\cdot {N}_{{\rm{0}}}$, the basal division rate *b*_0_, the variance of mutation effects *σ*, the range of mutation effects distribution ${r}_{max}/{b}_{{\rm{0}}}$, the mutation rate *μ*, and the frequency of mutations with NCA effects. PRCC were computed using a variant of function pcc (modified to deliver p-values) of the statistical software R (package sensitivity^39^).

To assess the effect of growth kinetics on CD regardless of NCA effects, we computed PRCC of Simpson index depending on minimal tumor size ${\alpha }_{t}\cdot {N}_{{\rm{0}}}$, the basal division rate *b*_0_, the variance of mutation effects *σ*, the range of mutation effects distribution ${r}_{max}/{b}_{{\rm{0}}}$, the mutation rate *μ*, and tumor growth rate. PRCC residuals of Simpson index and tumor growth rate were extracted to investigate for potential correlations with Pearson correlation coefficient.

## Results

### Impacts of NCA effects on clonal diversity

**Figure 2:**
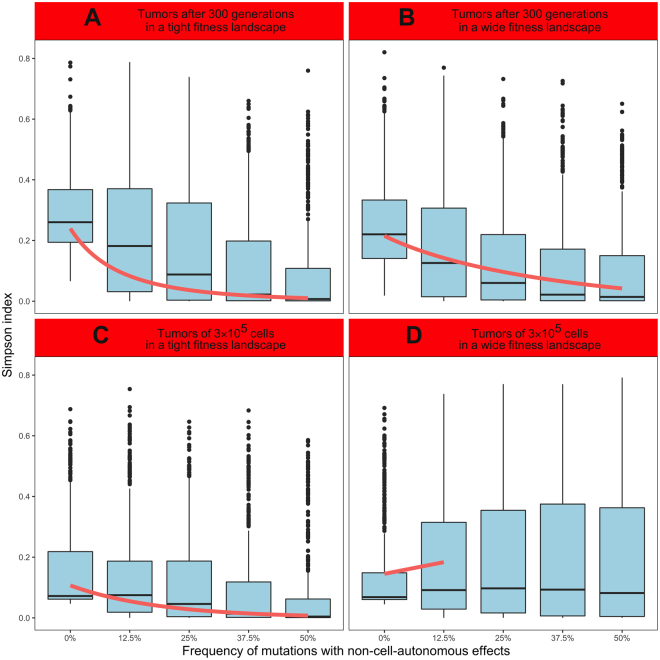
Impacts of NCA effects on tumor diversification. 500 simulations were performed for each condition with the following parameters: *N*_0_ = 10000, ${b}_{{\rm{0}}}={d}_{0}\in [{\rm{4}}{{\rm{.10}}}^{-{\rm{3}}},6{{\rm{.10}}}^{-{\rm{3}}}]$, *t*_*max*_ = 300, *N*_*max*_ *= 3*0 *N*_0_, ${r}_{max}\in [1.6\,{b}_{0},2.4\,{b}_{0}]$, *b*_*max*_ *= d*_*max*_ *= 5 r*_*max*_, $\mu \in [{\rm{8}}{{\rm{.10}}}^{-{\rm{5}}},1.2{{\rm{.10}}}^{-{\rm{4}}}]$, $\sigma \in [{\rm{0.8}},1.2]$ and ${\alpha }_{t}\in [{\rm{2.4}},3.6]$. Simpson index is displayed in light blue, and linear regressions in red. (**A**) Simulations performed during 300 generations for a tight fitness landscape (linear regression, R^2^ = 0.208). (**B**) Simulations performed during 300 generations for a wide fitness landscape (linear regression, R^2^ = 0.147). (**C**) Simulations performed until the tumor reaches 3.10^5^ cells for a tight fitness landscape (linear regression, R^2^ = 0.183). (**D**) Simulations performed until the tumor reaches 3.10^5^ cells for a wide fitness landscape (linear regression, R^2^ = 0.011).

We simulated 2500 tumors during 300 cell generations, in both a tight and a wide fitness landscape. In a tight fitness landscape, Simpson index decreases when the frequency of mutations with NCA effects increases (Fig. 2A, linear regression: $\mathrm{log}(Simpson\,index)=-{\rm{4.49}}\pm {\rm{0.17}}\cdot \sqrt{{f}_{NCA}}$, t = −25.62, p < 2.2.10^−16^, R^2^ = 0.208). The sensitivity analysis supports this negative significant association with the frequency of mutations with NCA effects (PRCC, r = −0.431, p < 2.2.10^−16^), as well as with the range of mutation effects (ranging from 1.6 *b*_0_ *= 1*.6 *d*_0_ to 2.4 *b*_0_ *= 2*.4 *d*_0_, r = −0.059, p = 3.4.10^−3^) and with minimal tumor size (ranging from 2.4 *N*_0_ to 3.6 *N*_0_, r = −0.051, p = 1.1.10^−2^, Supporting information, Table S3). For tumors simulated in a wide fitness landscape, we observed the same relationship: Simpson index is negatively associated with the frequency of mutations with NCA effects (Fig. 2B, linear regression: $\sqrt{Simpson\,index}=-{\rm{0.38}}\pm {\rm{0.02}}\cdot \sqrt{{f}_{NCA}}$, t = −20.78, p < 2.2.10^−16^, R^2^ = 0.147). Here again, the sensitivity analysis measured negative significant associations of Simpson index with the frequency of mutations with NCA effects (PRCC, r = −0.372, p < 2.2.10^−16^) as well as with the range of mutation effects (r = −0.041, p = 4.2.10^−2^, Supporting information, Table S3).

We also simulated 2500 tumors until they reached 3.10^5^ cells, in both fitness landscapes. In a tight fitness landscape, we also found a negative association of Simpson index with the frequency of mutations with NCA effects (Fig. 2C, linear regression: $\mathrm{log}(Simpson\,index)=-{\rm{5.33}}\pm {\rm{0.22}}\cdot {f}_{NCA}$, t = −23.69, p < 2.2.10^−16^, R^2^ = 0.183). The sensitivity analysis supports this relationship with the frequency of mutations with NCA effects (PRCC, r = −0.388, p < 2.2.10^−16^), but also measured a positive significant association with the range of mutation effects (r = 0.055, p = 6.0.10^−3^, Supporting information, Table S3). However, in a wide fitness landscape, Simpson index increases with the frequency of mutations with NCA effects between 0% and 12.5% of mutations with NCA effects (Fig. 2D, linear regression: $Simpson\,index={\rm{0.31}}\pm {\rm{0.09}}\cdot {f}_{NCA}$, t = 3.46, p = 5.7.10^−4^, R^2^ = 0.011). Above 12.5%, Simpson index does not significantly vary with the frequency of mutations with NCA effects. Though, the sensitivity analysis does not support this relationship, and measured a negative significant association of Simpson index with the frequency of mutations with NCA effects (PRCC, r = −0.057, p = 4.2.10^−3^). It also measured positive significant associations with the range of mutation effects (r = 0.052, p = 8.7.10^−3^), as well as the basal division rate (ranging from 4.10^−3^ to 6.10^−3^, r = 0.043, p = 3.1.10^−2^) and the mutation rate (ranging from 8.10^−5^ to 1.2.10^−4^, r = 0.046, p = 2.1.10^−2^, Supporting information, Table S3).

### Impacts of NCA effects on tumor growth kinetics

**Figure 3:**
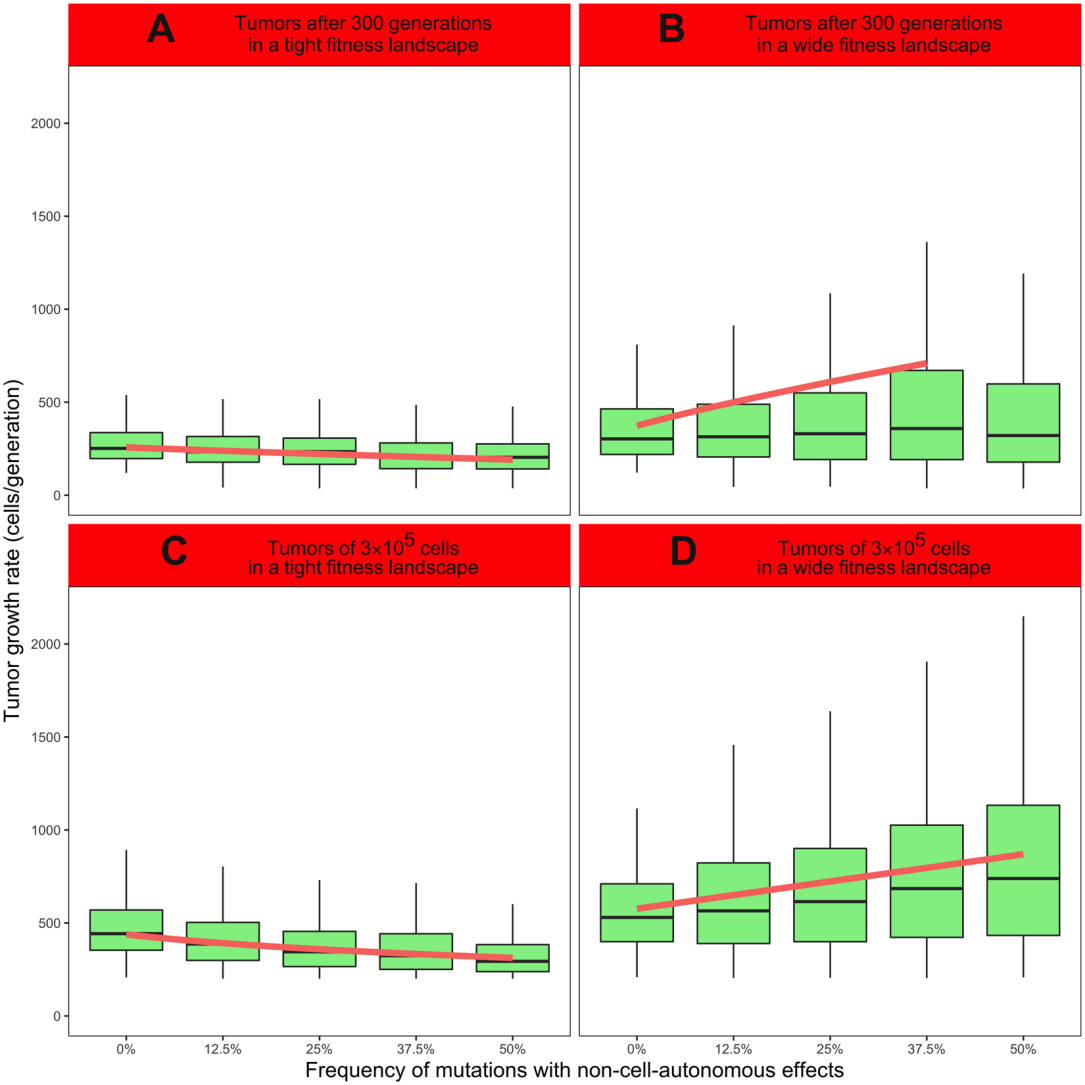
Impacts of NCA effects on tumor progression. 500 simulations were performed for each condition with the following parameters: *N*_*0*_ = 10000, ${b}_{{\rm{0}}}={d}_{0}\in [{\rm{4}}{{\rm{.10}}}^{-{\rm{3}}},6{{\rm{.10}}}^{-{\rm{3}}}]$, *t*_*max*_ = 300, *N*_*max*_ = *30 N*_*0*_, ${r}_{max}\in [1.6\,{b}_{0},2.4\,{b}_{0}]$, *b*_*max*_ = *d*_*max*_ = *5 r*_*max*_, $\mu \in [{\rm{8}}{{\rm{.10}}}^{-{\rm{5}}},1.2{{\rm{.10}}}^{-{\rm{4}}}]$, $\sigma \in [{\rm{0.8}},1.2]$ and ${\alpha }_{t}\in [{\rm{2.4}},3.6]$. Tumor growth rate is displayed in light green, and linear regressions in red. Outliers of tumor growth rate distribution are not displayed. (**A**) Simulations performed during 300 generations for a tight fitness landscape (linear regression, R^2^ = 0.046). (**B**) Simulations performed during 300 generations for a wide fitness landscape (linear regression, R^2^ = 0.026). (**C**) Simulations performed until the tumor reaches 3.10^5^ cells for a tight fitness landscape (linear regression, R^2^ = 0.109). (**D**) Simulations performed until the tumor reaches 3.10^5^ cells for a wide fitness landscape (linear regression, R^2^ = 0.057).

We measured tumor growth rate as an index of tumor growth kinetics for the tumors simulated in a tight fitness landscape. For tumors simulated during the same amount of time in a tight fitness landscape, tumor growth rate is negatively associated with the frequency of mutations with NCA effects increases (Fig. 3A, linear regression: $\mathrm{log}(Tumor\,growth\,rate)=-{\rm{0.59}}\pm {\rm{0.05}}\cdot {f}_{NCA}$, t = −11.02, p < 2.2.10^−16^, R^2^ = 0.046). The sensitivity analysis supports this negative association with the frequency of NCA effects (PRCC, r = −0.221, p < 2.2.10^−16^), but tumor growth rate is also significantly positively associated with minimal tumor size (r = 0.278, p < 2.2.10^−16^), as well as with the basal division rate (r = 0.178, p < 2.2.10^−16^) and the range of mutation distribution (r = 0.376, p < 2.2.10^−16^, Supporting information, Table S4). Conversely, in a wide fitness landscape, tumor growth rate is positively associated with the frequency of mutations with NCA effects (Fig. 3B, linear regression: $\sqrt{Tumor\,growth\,rate}={\rm{11.88}}\pm {\rm{1.60}}\cdot \sqrt{{f}_{NCA}}$, t = 7.41, p = 1.9.10^−13^, R^2^ = 0.026). Above 37.5%, tumor growth rate does not significantly vary with the frequency of mutations with NCA effects. This positive association with the frequency of mutations with NCA effects is confirmed by the sensitivity analysis (PRCC, r = 0.077, p = 1.1.10^−4^). The sensitivity analysis also measured significant positive associations with minimal tumor size (r = 0.175, p < 2.2.10^−16^), as well as with the basal division rate (r = 0.136, p = 7.4.10^−12^), and the range of mutation distribution (r = 0.146, p = 2.3.10^−13^, Supporting information, Table S4).

For tumors simulated until reaching the same size in a tight fitness landscape, tumor growth rate is nega‑tively associated with the frequency of mutations with NCA effects (Fig. 3C, linear regression: $\mathrm{log}(Tumor\,growth\,rate)=-{\rm{0.48}}\pm {\rm{0.03}}\cdot \sqrt{{f}_{NCA}}$, t = −17.53, p < 2.2.10^−16^, R^2^ = 0.109). The sensitivity analysis supports this negative relationship with the frequency of NCA effects (PRCC, r = −0.453, p < 2.2.10^−16^), and also measured significantly positive associations with minimal tumor size (r = 0.134, p = 2.1.10^−11^), the basal division rate (r = 0.184, p < 2.2.10^−16^), and the range of mutation distribution (r = 0.416, p < 2.2.10^−16^, Supporting information, Table S4). In a wide fitness landscape, tumor growth rate increases with the frequency of mutations with NCA effects (Fig. 3D, linear regression: $Tumor\,growth\,rate={\rm{585.42}}\pm {\rm{47.55}}\cdot {f}_{NCA}$, t = 12.31, p < 2.2.10^−16^, R^2^ = 0.057). This positive association with the frequency of mutations with NCA effects is confirmed by the sensitivity analysis (PRCC, r = 0.195, p < 2.2.10^−16^), which also measured significant positive associations with minimal tumor size (r = 0.128, p = 1.5.10^−10^), the basal division rate (r = 0.146, p = 2.3.10^−13^), and the range of mutation distribution (r = 0.117, p = 4.0.10^−9^, Supporting information, Table S4).

We globally measured a significant negative association between Simpson index and tumor growth rate when tumors were simulated in a wide fitness landscape, but no correlation for a tight fitness landscape (Supporting information, Table S5 and Fig. S2).

## Discussion

For a tight fitness landscape, *i*.*e*. when CA and NCA effects mix up to impact the division and death rates, frequent NCA effects are associated to lower CD (cf. Fig. 2A,C). For a wide fitness landscape, *i*.*e*. when NCA effects extend the range of potential impacts of mutations on clonal growth rates, the relationship between NCA effects and CD is the same only when tumors grow during a fixed number of generations (Fig. 2B). This negative relationship is confirmed by the sensitivity analysis, where the partial rank correlation between CD and the frequency of NCA effects is systematically negative (Supporting information, Table S3). We could also measure the opposite relationship (*i*.*e*., when frequent NCA effects yield higher CD, Fig. 2D) for tumors growing until a fixed size, but only for low frequencies (0 to 12.5%) of mutations with NCA effects, and in contradiction with the sensitivity analysis (Table S3). We suspected this latter positive relationship to depend on each tumor’s size: it was only observed when all the simulations (2500/2500) yielded tumors larger than 3.10^5^ cells, while the negative relationship could be observed for smaller tumors (*e*.*g*., Fig. 2B: 407/2500 simulations yielded tumors larger than 3.10^5^ cells). Yet, we still observed a negative relationship between NCA effects and CD when performing the same analysis on the sample of tumors larger than 3.10^5^ cells (Supporting information, Fig. S3). Moreover, tumors growing until a fixed size could evolve during a wider timeframe (88 to 1492 generations) than tumors growing during a fixed number of generations (300 generations): this configuration could render the positive relationship between CD and NCA effects risky to interpret. Thus, we will not discuss these results any further, and we suggest that NCA effects and CD should generally have a negative relationship, regardless of the fitness landscape considered. Besides, the relationship between tumor growth and NCA effects strongly depends on the fitness landscape: frequent NCA effects generate lower tumor growth rate in a tight fitness landscape (Fig. 3A,C, Supporting information, Table S4), but higher tumor growth rate in a wide fitness landscape (Fig. 3B,D, Supporting information, Table S4). Moreover, though we found no association between tumor growth rate and Simpson index for tumors simulated in a tight fitness landscape (Supporting information, Table S4 and Fig. S2A,C), high CD is associated with slow tumor growth kinetics in tumors simulated in a wide fitness landscape (Table S4 and Fig. S2B,D).

**Figure 4:**
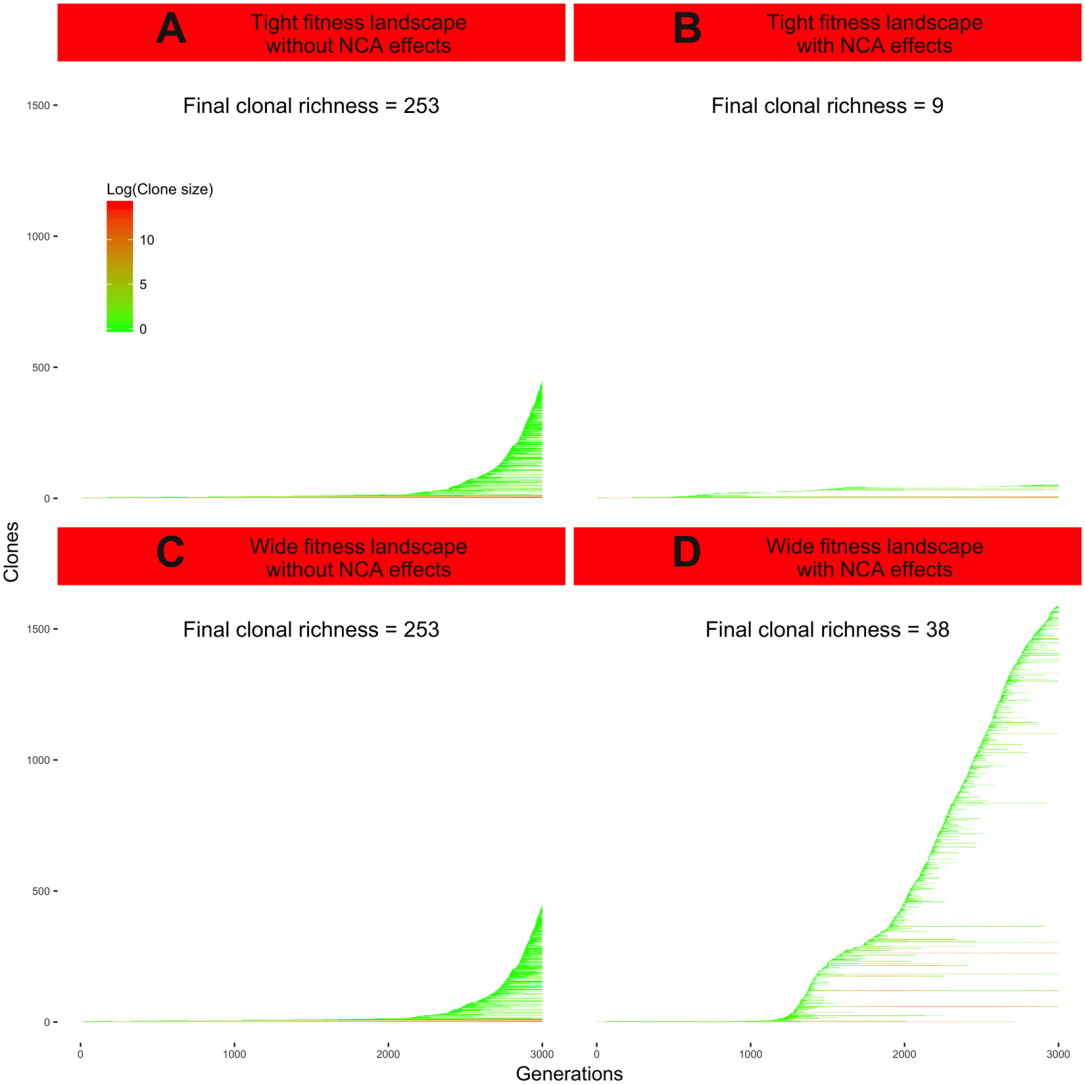
Emergence of new clones across time. Each panel displays the monitoring of a simulation during 3000 generations. (**A**) Simulation of branching evolution in a tumor with no NCA effect and a tight fitness landscape. (**B**) Simulation of branching evolution in a tumor with 100% of mutations with NCA effects and a tight fitness landscape. (**C**) Simulation of branching evolution in a tumor with no NCA effect and a wide fitness landscape. (**D**) Simulation of branching evolution in a tumor with 100% of mutations with NCA effects and a wide fitness landscape.

In a tight fitness landscape, CA effects can be the main component of clonal growth kinetics. Thus, neutral mutations arising in the fastest growing clones give birth to a large range of similarly growing clones that can coexist and maintain CD at a high level (cf. Figure 4A, considering that neutral mutations give rise to phenotypic changes that are unrelated to growth kinetics). This higher CD can be mitigated by the emergence of mutations with detrimental NCA effects, that can slow down clonal growth kinetics. Due to the tight bounds of the fitness landscape, these detrimental impacts are quite difficult to be balanced by beneficial NCA effects (cf. Fig. 4B). In this context, tumors are therefore likely to grow faster without NCA effects, and the contribution of NCA effects is therefore detrimental to CD.

In a wide fitness landscape, NCA effects can be the main component of clonal growth kinetics over the impacts of CA effects. In this context, many mutations are likely to shift the sensitivity of the latest clones to NCA effects. Similarly growing clones are therefore unlikely to coexist in this context, which significantly impairs cooperation and promotes competition between clones. Thus, beneficial NCA effects can not only support the growth of predominant clones already benefitting from their beneficial CA effects, but also allow clones undergoing their detrimental CA effects to persist in the tumor. Similarly, detrimental NCA effects can both slow down the growth of the predominant clones and speed up the extinction of the slowest growing ones (cf. Fig. 4D). In this context, we found a negative relationship between CD and tumor growth kinetics, which could indicate that NCA effects mainly drive the growth of predominant clones (cf. Fig. 4D), thus lowering CD.

Our model predicts not only that NCA effects do not drive tumors to high CD, but also that they impair the increase of CD, by shaping the growth rates of preexisting and emerging clones. This pattern is clearly not expected, considering that all previous observations and discussions on the impacts of NCA effects on CD have supported the hypothesis that NCA effects should promote CD^20–23, 25, 40–44^. However, these results have been observed either *in vitro* ^44^ or in tumor grafts or xenografts^20–22, 40–43^, and thus have not embraced the whole complexity of possible interactions within a tumor^25, 45^. These studies have simultaneously considered two clones carrying mutations with NCA effects or less, most of the time in established tumors. Conversely, our model theoretically allows considering up to 50% of clones carrying mutations with NCA effects among large mixtures of clones, in tumors that expand unrestrictedly. Therefore, though NCA effects may support coexistence for a few clones in established tumors, our results tend to show that this would not be the case when larger assemblages emerge over a short time interval, as is common during carcinogenesis^36, 46, 47^. It is yet to note that our results only regard early (because small-sized), expanding tumors (or expanding tumor niches), and that our model may yield different results for larger cancer cell populations and/or higher numbers of generations.

These differences between our results and previous observations could be explained by an increase of competition when NCA effects are very frequent. Indeed, by shaping the growth kinetics of various clones, CA and NCA effects contribute to establish a network of biological interactions between clones^21, 44^. Biological interactions have been widely studied in ecology and evolution to explain the respective impacts of different species on each other’s growth^48^, ranging from competition (mutually detrimental) to mutualism (mutually beneficial), and their impacts on cancer progression have been increasingly pointed out due to the many similarities between tumors and wild ecosystems^23, 25, 45, 49^. Here, detrimental NCA effects may be considered as interference competition, since cancer cells can remotely suppress each other’s growth^50^. Competition events were shown to yield a drop of CD^19^. On the contrary, mutualistic and commensalistic interactions, which can be involved through beneficial NCA effects, can promote the coexistence of several clones^20, 41, 42, 44, 49, 51^. Though, when beneficial NCA effects depend on the relative abundances of clones (which is not the case here), they behave like limited resources and can result in other forms of exploitation competition^21, 44, 51, 52^.

The relationship that we found between NCA effects, clonal growth kinetics and CD can be put in perspective of clinical data. Indeed, our results forecast that, when NCA effects have distinct impacts on clonal growth rates from CA effects’, the slowest growing tumors should be the most clonally diverse after a given amount of time. Indeed, when clinically detected, tumors mostly composed of slowly-dividing cancer cells are generally described as heterogeneous^9^ and aggressive^53^, while fast-growing tumors usually well respond to treatments^54^. However, the link between tumor growth kinetics and ITH was mostly established for tumors of clinical size, often after several years under various regimens of selection^55^, and our results predict these heterogeneous, slow growing tumors to occur when mutations with NCA effects are rare. This could be the reason why so few mutations with NCA effects have been described so far. Moreover, if NCA effects may have a limited contribution to growth kinetics of clinical-stage tumors, there are evidence that NCA effects can significantly contribute to early carcinogenesis^29, 56, 57^. Further studies should therefore investigate tumor growth kinetics at early stages, in order to clearly identify the contribution of NCA effects to ITH.

The design of this model mainly aimed at providing a framework to investigate the underappreciated impact of NCA effects on ITH^58^. Though, for the sake of simplicity, we made several assumptions that deserve to be discussed and further investigated. In this model, we assumed that divisions always occurred before death events. However, the order of birth and death events may have large impacts on the outcome of discrete eco-evolutionary processes (especially in the evolution of interactions)^59^, and results might thus differ for further studies considering other division and death orders. Morever, the contribution of NCA effects to a clone’s growth kinetics do not depend on any clone’s size: *i*.*e*., NCA effects are not limiting factors. Some NCA effects rely on widely secreted metabolites^50, 60–62^ or on the extensive recruitment of stromal cells^21, 63^, and should be well described by this model. Others might be under frequency-dependent selection^20, 44^, but their incidence in tumors has not been established. Furthermore, we assumed that factors involving NCA effects (*e*.*g*., growth factors, miRNAs) diffuse instantly from one cell to another, which is rarely encountered *in vitro* and *in vivo* ^28, 44, 61^. Diffusion constraints should play a significant role in shaping CD, either directly by selecting a range of interacting partners^44^, or indirectly by shaping the growth kinetics^28^, or even by allowing the construction of spatially distinct niches simultaneously dominated by different clones^23^. Though, this model only encompasses up to 10^7^ cells (see section 2.2) at the same time, which should correspond to 0.01–0.1 mm^3^ *in vivo*:^64^ we can therefore easily assume that the diffusion time is negligible before the average cell cycle duration. Stromal cells involved in some NCA effects should also spread quickly at such scales^21, 63^. Besides, tumors are very spatially heterogeneous^65^ and the ME usually consists of several niches that may sparsely interfere with each other, so that we can easily assume this model to describe the cell dynamics at stake in the local ME. Tumors of clinic size actually gather several billions of cells in average^64, 66^, and this model could thus be nested in wider-scale models of tumor dynamics. Finally, it is to note the impacts of NCA effects on tumor growth kinetics have been very sparsely quantified. Thus, we do not know whether up to 50% of mutations arising during cancer progression might have NCA effects, which is something crucial to quantify.

Since ITH is usually a good proxy for cancer aggressiveness and post-therapeutic recurrence at clinical stages^2, 4–7^, a major challenge in cancer research is to better predict the evolution of phenotypic diversity in a patient’s tumor. In this study, we have pointed out the possibility to identify a range of selection regimens during tumor growth, in which various mutational landscapes contribute to shape CD in the tumor. Notably, we found that when NCA effects made little contribution to growth kinetics, CD was the highest for the fastest growing tumors. Conversely, when NCA effects made significant contribution to growth kinetics, CD was the highest for the slowest growing tumors. Such theoretical predictions could allow new perspectives in the prediction of each patient’s ITH. Indeed, ITH is well-documented for some cancers for which aggressiveness strongly depends on phenotypic diversity (e.g. glioblastoma multiforme^14, 67^), and growth kinetics of such cancers are monitored for clinical stages. Nevertheless, though their contributions to shaping the ME are widely studied, the emergence of mutations with NCA effects is still poorly understood and should call for a systematic identification of these mutations in order to predict the tumor’s outcome.

### Data availability statement

No datasets were generated or analysed during the current study.

## Electronic supplementary material

Supporting information
