# Supplementary material for: Non-cell-autonomous effects yield lower clonal diversity in expanding tumors
Source: Sci Rep. 2017 Sep 11;7:11157. doi: 10.1038/s41598-017-11562-w (PMC5593982; doi:10.1038/s41598-017-11562-w)
Supplement: Supplementary file 1 — Supporting information [file 41598_2017_11562_MOESM1_ESM.pdf]

Supporting information for:

Non-cell-autonomous effects yield lower clonal diversity in expanding tumors.

Tazzio Tissot[1,*], Frédéric Thomas[1], Benjamin Roche[1,2]

1- CREEC/MIVEGEC, UMR IRD/CNRS/UM 5290, 911 Avenue Agropolis, BP 64501, 34394 Montpellier Cedex 5, France

2- Unité mixte internationale de Modélisation Mathématique et Informatique des Systèmes Complexes. (UMI IRD/UPMC UMMISCO), 32 Avenue Henri Varagnat, 93143 Bondy Cedex, France

*corresponding author: tazziotissot@gmail.com

This section gathers complementary references supporting our model and parameters, as well as complementary analyses of the simulations and complementary information on the model architecture. Tables S1 and S2 respectively expose a nonexhaustive review of non-cell-autonomous (NCA) effects impacting growth rates in tumors, and the list of parameters that were chosen in our model. We performed sensitivity analysis on Simpson index and tumor growth rate by computing Partial Rank Correlation Coefficients, which are displayed in Tables S3 and S4. We performed univariate linear regressions on PRCC residuals for Simpson index as a function of tumor growth rate, which are displayed in Table S5. Figure S1 shows the impacts of NCA effects on clonal diversity measured as Shannon index. The predictions of the linear regressions displayed in Tab. S5 are showed with residuals in Figure S2. Figure S3 shows the impacts of NCA effects on Simpson index for a subset of the largest tumors of Fig. 2B. Texts S1 and S2 expose respectively a detailed description and a simplified algorithm for the model.

Table S1: Examples of non-cell-autonomous effects impacting the growth of noncarrier clones

Type of NCA effect	Mechanism	Impacted cells	Effect on growth	Type of tumor
**Release of growth factors**	Acidic Fibroblast Growth Factor	All	+	*In vivo* mixture of carcinoma cells[9]
	Prostaglandin E2	Cancer stem cells	+	Human bladder cancer xenograft[10]
	Interleukin 6	High wtEGFR cells	+	Glioblastoma multiforme[11]
	Wnt1	Basal ΔHras cells	+	Mouse breast cancer model[12]
	Upd cytokines	Ras^{V12} cells	+	Fruitfly eye-antennal disc cancer[13]
	Insulin-like Growth Factor II	All	+	Neuroendocrine pancreatic cancer cells[14]
**Release of miRNAs**	Cell-independently processed miRNAs	Nontumorigenic epithelial cells	+	Breast cancer[15]
**Secretion of metabolites**	Lactic acid, ATP-H^+ pump	All but resistant cells	-	Human breast cancer among others[16–18]
	Hyperlipidemia induced by Very Low-Density Lipoprotein secretion	All	+	Subcutaneous mouse tumor model including Bcr/Abl-transformed precursor B cells[1]
**Remodeling of the local ME**	Matrix Metalloproteinase 14	All	+	Zebrafish-melanoma xenograft[2]
	Vasculogenic mimicry	All	+	Human melanoma, mouse breast cancer model[3,4]
**Recruitment of stromal cells**	Endothelial cells recruited by Vascular Endothelial Growth Factor	All	+	Mouse pancreatic cancer[5]
	Various stromal cells recruited by Interleukin 6	All	+	Mouse breast cancer xenograft[6]
**Unknown**	Unknown	All	-	Mouse mammary cancer, subcutaneous rat tumor model[7,8]

Table S2: Parameter values of the model

Parameter	Value	80% Value	120% Value
N_0	10000[19]		
$b_0 = d_0$	5.10^{-3} [6,20]	4.10^{-3}	6.10^{-3}
t_{max}	300[21]		
N_{max}	$30\,N_0$[19]		
r_{max}	$2\,b_0 = 2\,d_0$[20]	$1.6\,b_0 = 1.6\,d_0$	$2.4\,b_0 = 2.4\,d_0$
$b_{max} = d_{max}$	$5\,r_{max}$[20]		
μ	1.10^{-4} [21,22]	8.10^{-5}	$1.2.10^{-4}$
σ	1	0.8	1.2
$\alpha_t\,N_0$	$3\,N_0$	$2.4\,N_0$	$3.6\,N_0$

Table S3: Sensitivity analysis of Simpson index

Variables		Minimal tumor size $\alpha_t\,N_0$	Basal division rate b_0	Variance of mutation effects σ	Range of mutation effects distribution r_{max}/b_0	Mutation rate μ	Frequency of mutations with NCA effects
**Tumors of same age Tight fitness landscape**	**PRCC**	-0.051	$-6.7.10^{-4}$	-0.011	-0.059	-0.008	-0.431
	**p**	$1.1.10^{-2}$	$9.7.10^{-1}$	$6.0.10^{-1}$	$3.4.10^{-3}$	$7.0.10^{-1}$	$<2.2.10^{-16}$
**Tumors of same age Wide fitness landscape**	**PRCC**	-0.019	-0.009	-0.001	-0.041	0.012	-0.372
	**p**	$3.2.10^{-1}$	$6.4.10^{-1}$	$9.5.10^{-1}$	$4.2.10^{-2}$	$5.4.10^{-1}$	$<2.2.10^{-16}$
**Tumors of same size Tight fitness landscape**	**PRCC**	-0.004	-0.029	0.006	0.055	0.031	-0.388
	**p**	$8.4.10^{-1}$	$1.4.10^{-1}$	$7.5.10^{-1}$	$6.0.10^{-3}$	$1.2.10^{-1}$	$<2.2.10^{-16}$
**Tumors of same size Wide fitness landscape**	**PRCC**	0.002	0.043	-0.010	0.052	0.046	-0.057
	**p**	$9.0.10^{-1}$	$3.1.10^{-2}$	$6.2.10^{-1}$	$8.7.10^{-3}$	$2.1.10^{-2}$	$4.2.10^{-3}$

Table S4: Sensitivity analysis of tumor growth rate

Variables		Minimal tumor size $\alpha_t N_0$	Basal division rate b_0	Variance of mutation effects σ	Range of mutation effects distribution r_{max}/b_0	Mutation rate μ	Frequency of mutations with NCA effects
**Tumors of same age Tight fitness landscape**	**PRCC**	0.278	0.178	0.003	0.376	-0.013	-0.221
	**p**	$<2.2.10^{-16}$	$<2.2.10^{-16}$	$8.8.10^{-1}$	$<2.2.10^{-16}$	$5.1.10^{-1}$	$<2.2.10^{-16}$
**Tumors of same age Wide fitness landscape**	**PRCC**	0.175	0.136	$-1.9.10^{-4}$	0.146	0.023	0.077
	**p**	$<2.2.10^{-16}$	$7.4.10^{-12}$	$9.9.10^{-1}$	$2.3.10^{-13}$	$2.5.10^{-1}$	$1.1.10^{-4}$
**Tumors of same size Tight fitness landscape**	**PRCC**	0.134	0.184	0.015	0.416	-0.007	-0.453
	**p**	$2.1.10^{-11}$	$<2.2.10^{-16}$	$4.4.10^{-1}$	$<2.2.10^{-16}$	$7.1.10^{-1}$	$<2.2.10^{-16}$
**Tumors of same size Wide fitness landscape**	**PRCC**	0.128	0.146	0.001	0.117	0.034	0.195
	**p**	$1.5.10^{-10}$	$2.3.10^{-13}$	$9.6.10^{-1}$	$4.0.10^{-9}$	$8.8.10^{-2}$	$<2.2.10^{-16}$

Table S5: Univariate linear regressions for PRCC residuals of Simpson index as a function of PRCC residuals of tumor growth rate.

PRCC residuals of Simpson index ~ PRCC residuals of tumor growth rate	Estimate	Standard error	Intercept	Standard error	R^2
Tumors of same age – Wide fitness landscape	$-1.861.10^{-1}$	$2.038.10^{-2}$	$1.105.10^{-14}$	14.19	0.032
Tumors of same size – Wide fitness landscape	$-4.082.10^{-1}$	$1.877.10^{-2}$	$-9.891.10^{-14}$	13.20	0.159

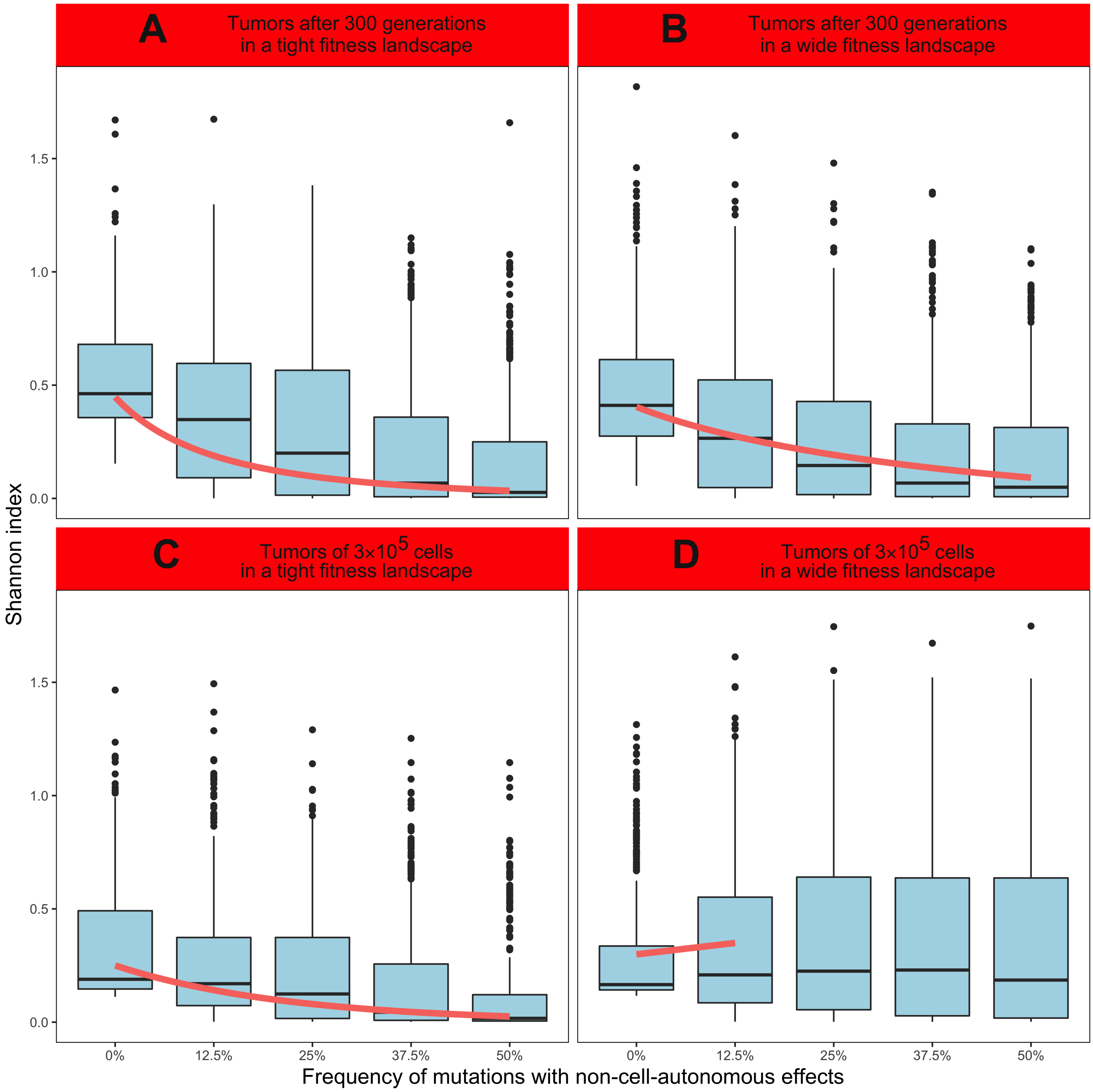

Figure S1: Impacts of NCA effects on tumor diversification measured with Shannon index. Each panel displays the result of $5 * 500 = 2500$ simulations. Shannon index is displayed in light blue, and linear regressions in red. (A) Simulations performed during 300 generations for a tight fitness landscape (linear regression, R^2=0.209). (B) Simulations performed during 300 generations for a wide fitness landscape (linear regression, R^2=0.152). (C) Simulations performed until the tumor reaches 3.10^5 cells for a tight fitness landscape (linear regression, R^2=0.200). (D) Simulations performed until the tumor reaches 3.10^5 cells for a wide fitness landscape (linear regression, R^2=0.006). Parameters used are displayed in Table S2.

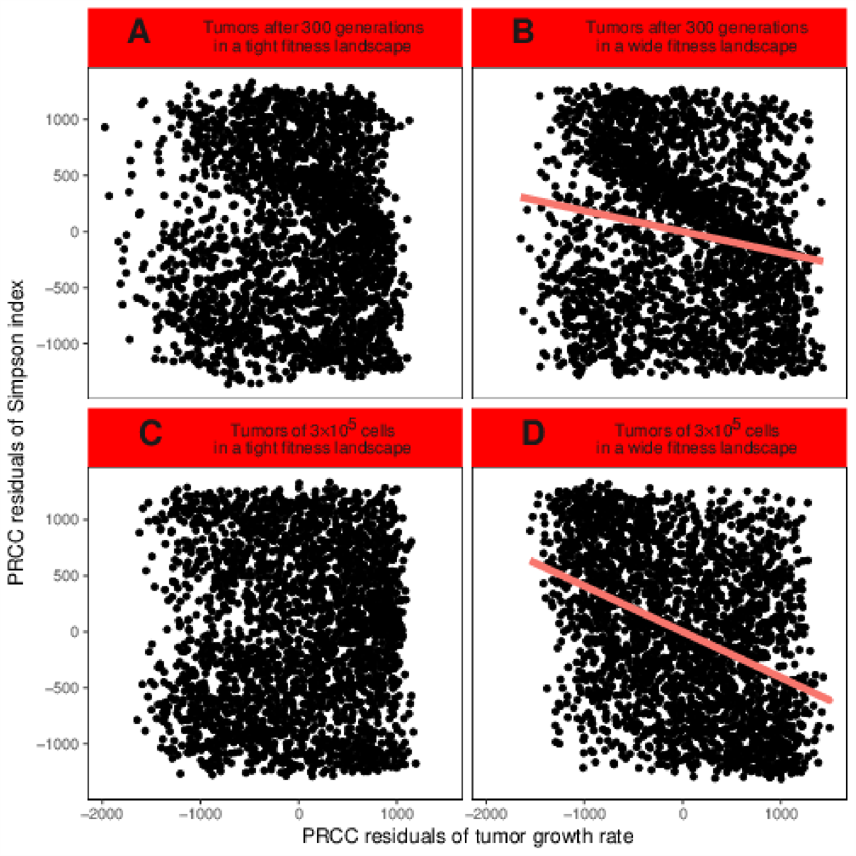

Figure S2: Relationship between clonal diversity and tumor growth kinetics. Each panel displays the result of $5 * 500 = 2500$ simulations. Residuals are displayed as black dots and the linear models built in Tab. S3 are displayed as red lines. (A) PRCC residuals of Simpson index as a function of PRCC residuals of tumor growth rate after 300 generations with a tight fitness landscape. (B) PRCC residuals of Simpson index as a function of PRCC residuals of tumor growth rate after 300 generations with a wide fitness landscape (linear regression, R^2=0.032). (C) PRCC residuals of Simpson index as a function of PRCC residuals of tumor growth rate for 3.10^5 cells with a tight fitness landscape. (D) PRCC residuals of Simpson index as a function of PRCC residuals of tumor growth rate for 3.10^5 cells with a wide fitness landscape (linear regression, R^2=0.159). Parameters used are displayed in Table S2.

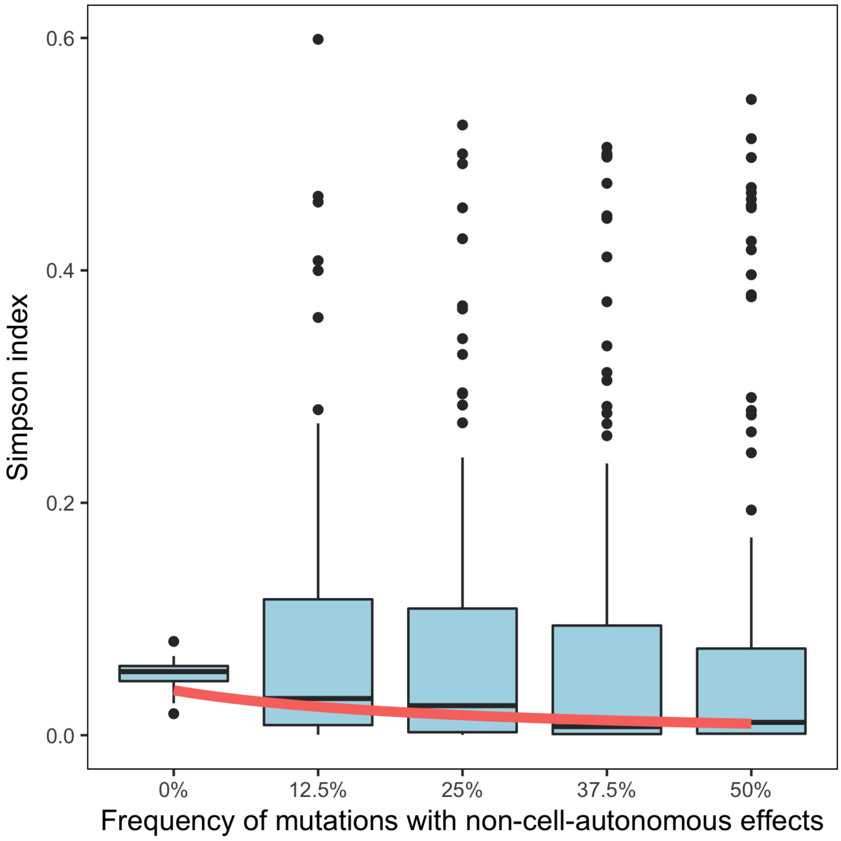

Figure S3: Impacts of non-cell-autonomous effects on clonal diversity for the largest tumors after 300 generations in a wide fitness landscape. This figure is a subset of Fig. 2B (tumors after 300 generations in a wide fitness landscape), for tumors which size is greater than 3.10^5 cells. This subset gathers 407 simulations: 22 for f_{NCA}=0%, 65 for f_{NCA}=12.5%, 98 for f_{NCA}=25%, 107 for f_{NCA}=37.5%, and 115 for f_{NCA}=50%. Simpson index is displayed in blue, and a linear regression in red. This linear regression shows a significant negative association between the frequency of mutations with NCA effects and Simpson index (linear regression: $Log\,(\text{Tumor growth rate})=$ $-2.24\pm0.62\sqrt{f_{NCA}}$, t=-3.599, p=$3.6.10^{-4}$, R^2=0.029).

Text S1: Model description

S1.1 Classes and methods

We consider only one class of individual cells, belonging to diverse clones, and upon which we apply events of division, mutation and death. The probability of division and death events are calculated by applying an exponential distribution on each rate: P = 1 – *exp*(- *rate* x δ_t) where δ_t is the model integration time step and *rate* is the individual rate expressed in the mean field approximation. The probability of mutation events is calculated by applying the exponential distribution on mutation rate μ. Division, mutation and death events then occur stochastically according to the computed probabilities.

Each clone is associated to a vector ("mutationFitnessEffects") which size corresponds to the number of clones having emerged during previous generations. This vector contains values corresponding to effects of mutations on every clone's growth (genetically encoded traits). We consider that mutations carried by a clone can have effects both on this clone's growth (i.e. CA effects) and on other clones' growth (i.e. NCA effects).

S1.2 Program scheduler

Division, mutation and death events occur at discrete time, thereafter called "generations". Two steps are generally considered at each generation. First, we determine the offset of each clone according to the number of divisions and deaths that have occurred for each clone during the time step. Then, when all clones have computed their next offset, their size is updated through a second function. This two-steps method is important to avoid synchronization issues.[23]

In our case, at each generation, a function "Step" is called for every cell for clones which size ("cloneSize") is above zero. If a cell divides during this function, a clone attribute ("offsetCloneSize") will be incremented of one unit. If a cell dies during this function, "offsetCloneSize" is decremented of one unit. If a cell mutates during this function, "offsetCloneSize" is decremented of one unit, and another clone attribute ("newMutations") is incremented of one unit.

S1.3 Division and death processes

Within the “Step” function, division, mutation and death processes take place (see Fig. 4). For each cell present in the system at this time step, a division rate and a death rate are computed thanks to basal rates (b_0, d_0) and mutations' fitness effects (r_{ij} with (i, j) in [1,n]). For each individual, the division and death rates are computed as the basal division and death rates. The values of “mutationFitnessEffects” are tested whether positive or negative for each carrier clone present in the system (i.e. which size is strictly positive) during the generation: then, positive values are added to the division rate of the recipient individual, and negative values to its death rate. We limit the distribution of division and death rates in order not to obtain a superexponential growth;[20] thus we retain their computations between defined boundaries.

Then, for each clone i, the probabilities of division and death are computed with the previously computed division and death rates: the probability of division and death are equal to:

$$P^i_{division} = 1 - exp(-(b_0 + \sum_j b_{ji})\, \delta_t)$$
$$P^i_{death} = 1 - exp(-(d_0 + \sum_j d_{ji})\, \delta_t)$$

with $b_{ji} = r_{ji}$ if $r_{ji} \geq 0$ and $d_{ji} = r_{ji}$ if $r_{ji} < 0$. Division and then death events are selected according to these probabilities. During each division event, mutation events are selected according to the probability $P^i_{mutation} = 1 - exp(-\mu\, \delta_t)$.

After all cells have called the “Step” function, “cloneSize” is updated by adding to it the number contained in “offsetCloneSize”. Depending on “cloneSize” variations, we create new cells or destroy existing cells. If the updated “cloneSize” is below zero, it is raised to zero and the corresponding clone is then considered as extinct and no longer part of the tumor.

S1.4 Mutations

For each clone, we select as many mutation events as there are units in “newMutations”. For each mutation event, we first create a new cell that we consider to be part of the descending clone (see fig. 1B). We then create a new vector “mutationEffects” based on the ancestral clone's vector: we draw its descending trait values in normal distributions centered on ancestral trait values, and bounded by two pre-determined limits. We also append all clone's vectors with a new trait value

drawn in normal distributions centered on the values of the effects on the ancestral clone. We also set boundaries to every distribution, still in order to maintain an exponential cell growth.

For each mutation event, there is a given probability (corresponding to 1 minus the pre-determined frequency of NCA effects) that the mutation has only CA effects, and no NCA effect. Then, we set to zero all the values of “mutationEffects” but the clone's effect on itself.

Text S2: Model algorithms

S2.1 Class model

```
VOID GO()
        BEGIN
                std::vector<Individual> lTableIndividual;
                FOR(int lIndex=0; lIndex<nbCellInit; lIndex++)
                        Individual lTemp;
                        lTemp.setClone(0);
                        lTemp.setDivision(basalDivision);
                        lTemp.setDeath(basalDeath);
                        lTableIndividual.push_back(lTemp);
                END FOR
                abundanceClone.push_back(nbCellInit);
                mutantClone.push_back(0);
                offsetAbundanceClone.push_back(0);
                matrixEffect[0].push_back(0);
                double t=0;
                WHILE (t<tMax AND lTableIndividual.size()>0 AND lTableIndividual.size()<criticalSize)
                        //For each cell, call step function
                        FOR (lint IndexInd=0;lIndexInd<lTableIndividual.size();lIndexInd++)
                                lTableIndividual[lIndexInd].step(t);
                        END FOR
                        //Update each clone size
```

```
FOR (int lIndex=0;lIndex<offsetAbundanceClone.size();lIndex++)
        //For each clone, creation of new cells if the demographic balance is positive
        IF (offsetAbundanceClone[lIndex]>0)
                FOR (int lIndexNew=0;lIndexNew<offsetAbundanceClone[lIndex];lIndexNew++)
                        Individual lTemp;
                        lTemp.setClone(lIndex);
                        lTemp.setDivision(basalDivision);
                        lTemp.setDeath(basalDeath);
                        lTableIndividual.push_back(lTemp);
                END FOR
        END IF
        //For each clone, destruction of the first cells if the demographic balance is negative
        IF (offsetAbundanceClone[lIndex]<0)
                int lIndRemoved=0;
                int lIndexInd=0;
                WHILE (lIndexInd<lTableIndividual.size() AND lIndRemoved<(-offsetAbundanceClone[lIndex]))
                        IF (lTableIndividual[lIndexInd].getClone()==lIndex)
                                lTableIndividual.erase(lTableIndividual.begin()+lIndexInd);
                                lIndRemoved++;
                        ELSE
                                lIndexInd++
                        END IF
                END WHILE
```

```
END IF
abundanceClone[lIndex]+=offsetAbundanceClone[lIndex];
//Creation of new clones for mutant cells
double nbMutant=0;
int lIndexInd=0;
WHILE (nbMutant<mutantClone[lIndex])
        IF (lTableIndividual[lIndexInd].getClone()==lIndex)
                //Mutation event
                mutTrait.push_back(functionMutation(mutTrait[lIndex],lVariance,boundInf,boundSup));
                std::vector<double> lTemp;
                IF (randomUniform()<rateNCAMutation)
                        FOR (int lIndexClone=0;lIndexClone<matrixEffect.size();lIndexClone++)
                                lTemp.push_back(functionMutation(matrixEffect[lIndex][lIndexClone],lVariance,-1,1));
                        END FOR
                        matrixEffect.push_back(lTemp);
                        FOR (int lIndexClone=0;lIndexClone<matrixEffect.size()-1;lIndexClone++)
                                matrixEffect.push_back(functionMutation(matrixEffect[lIndexClone][lIndex],lVariance,-1,1));
                        END FOR
                        matrixEffect[matrixEffect.size()-1].push_back(functionMutation(matrixEffect[lIndex][lIndex],lVariance,-1,1));
                ELSE
                        FOR (int lIndexClone=0;lIndexClone<matrixEffect.size();lIndexClone++)
                                lTemp.push_back(0);
                        END FOR
```

```
                        matrixEffect.push_back(lTemp);
                        FOR (int lIndexClone=0;lIndexClone<matrixEffect.size();lIndexClone++)
                            matrixEffect.push_back(0);
                        END FOR
                        matrixEffect[matrixEffect.size()-1][matrixEffect.size()-1]=functionMutation(matrixEffect[lIndex][lIndex],lVariance,-1,1));
                    END IF
                    abundanceClone.push_back(1);
                    abundanceClone[lIndex]=abundanceClone[lIndex]-1;
                    offsetAbundanceClone.push_back(0);
                    mutantClone.push_back(0);
                    lTableIndividual[lIndexInd].setClone(abundanceClone.size());
                    nbMutant++;
                END IF
                lIndexInd++
            END WHILE
            //Resetting the demographic balance and the number of mutants
            offsetAbundanceClone[lIndex]=0;
            mutantClone[lIndex]=0;
        END FOR
        //Incrementing time
        t++;
    END WHILE
END
```

```
DOUBLE FUNCTIONMUTATION(DOUBLE valueToMutate, float lVariance, double boundInf, double boundSup)
    BEGIN
        double x1, x2;
        double finalValue;
        DO
            normalize(&x1,&x2);
            finalValue = x1*lVariance + valueToMutate;
        END DO WHILE (finalValue>boundSup OR finalValue<boundInf)
        RETURN finalValue;
    END

VOID NORMALIZE(double *x1, double *x2)
    BEGIN
        double W, V1, V2;
        DO
            double U1=randomUniform();
            double U2=randomUniform();
            V1=2*U1-1;
            V2=2*U2-1;
            W=V1*V1+V2*V2;
        END DO WHILE (W>1)
        double W_function=SQRT(-2*LOG(W)/W);
```

```
		*x1=V1*W_function;
		*x2=V2*W_function;
	END

DOUBLE RANDOMUNIFORM()
	BEGIN
		RETURN rand()/(double)RAND_MAX;
	END
```

S2.1 Class Individual

```
VOID INDIVIDUAL::STEP(float t)
	BEGIN
		float lProbaDivision=convertRateProbability(functionDivision(t));
		IF (lProbaDivision>randomUniform())
			offsetAbundanceClone[currentClone]++;
			float lMutationProb = 1-EXP(-mutationRate);
			IF (lMutationProb>randomUniform())
				mutationClone[currentClone]++;
			END IF
		END IF
		float lProbaDeath=convertRateProbability(functionDeath(t));
		IF (lProbaDeath>randomUniform())
			offsetAbundanceClone[currentSousPop]--;
```

```
        END IF
    END

FLOAT INDIVIDUAL::FUNCTIONDIVISION(float t)
    BEGIN
        float actualDivision=0;
        actualDivision+=division;
        int lIndex=0;
        WHILE (actualDivision<divisionBound AND lIndex<abundanceClone.size())
            double divisionTemp=mutTrait[lIndex]*matrixEffect[lIndex][currentClone];
            IF (divisionTemp>0 AND abundanceClone[lIndex]>0)
                actualDivision+=divisionTemp;
            END IF
            lIndex++;
        END WHILE
    END

FLOAT INDIVIDUAL::FUNCTIONDEATH(float t)
    BEGIN
        float actualDeath=0;
        actualDeath+=death;
        int lIndex=0;
        WHILE (actualDeath>deathBound AND lIndex<abundanceClone.size())
```

```
                double deathTemp=mutTrait[lIndex]*matrixEffect[lIndex][currentClone];
                IF (deathTemp<0 AND abundanceClone[lIndex]>0)
                    actualDeath+=deathTemp;
                END IF
                lIndex++;
            END WHILE
        END

VOID INDIVIDUAL::SETDIVISION(double pDivision)
        BEGIN
            division=pDivision;
        END

VOID INDIVIDUAL::SETDEATH(double pDeath)
        BEGIN
            death=pDeath;
        END
INDIVIDUAL::INDIVIDUAL(int pClone)
        BEGIN
            clone=pClone;
        END
INT INDIVIDUAL::GETCLONE()
        BEGIN
```

```
        RETURN clone;
    END

FLOAT INDIVIDUAL::CONVERTRATEPROBABILITY(double pRate)
    BEGIN
        RETURN 1-EXP(-pRate);
    END
```

Supporting Information References

1. Huang, J. *et al.* Tumor-Induced Hyperlipidemia Contributes to Tumor Growth. *Cell Rep.* **15,** 336–348 (2016).

2. Chapman, A. *et al.* Heterogeneous Tumor Subpopulations Cooperate to Drive Invasion. *Cell Rep.* **8,** 688–695 (2014).

3. Maniotis, A. J. *et al.* Vascular channel formation by human melanoma cells in vivo and in vitro: vasculogenic mimicry. *Am. J. Pathol.* **155,** 739–752 (1999).

4. Wagenblast, E. *et al.* A model of breast cancer heterogeneity reveals vascular mimicry as a driver of metastasis. *Nature* (2015). doi:10.1038/nature14403

5. Bergers, G. *et al.* Matrix metalloproteinase-9 triggers the angiogenic switch during carcinogenesis. *Nat. Cell Biol.* **2,** 737–744 (2000).

6. Marusyk, A. *et al.* Non-cell-autonomous driving of tumour growth supports sub-clonal heterogeneity. *Nature* **514,** 54–58 (2014).

7. Miller, B. E., Miller, F. R., Leith, J. & Heppner, G. H. Growth Interaction in Vivo between Tumor Subpopulations Derived from a Single Mouse Mammary Tumor. *Cancer Res.* **40,** 3977–3981 (1980).

8. Caignard, A., Martin, M. S., Michel, M. F. & Martin, F. Interaction between two cellular subpopulations of a rat colonic carcinoma when inoculated to the syngeneic host. *Int. J. Cancer* **36,** 273–279 (1985).

9. Jouanneau, J., Moens, G., Bourgeois, Y., Poupon, M. F. & Thiery, J. P. A minority of carcinoma cells producing acidic fibroblast growth factor induces a community effect for tumor progression. *Proc. Natl. Acad. Sci. U. S. A.* **91,** 286–290 (1994).

10. Kurtova, A. V *et al.* Blocking PGE2-induced tumour repopulation abrogates bladder cancer chemoresistance. *Nature* **517,** 209–213 (2015).

11. Inda, M.-M. *et al.* Tumor heterogeneity is an active process maintained by a mutant EGFR-induced cytokine circuit in glioblastoma. *Genes Dev.* **24,** 1731–1745 (2010).

12. Cleary, A. S., Leonard, T. L., Gestl, S. A. & Gunther, E. J. Tumour cell heterogeneity maintained by cooperating subclones in Wnt-driven mammary cancers. *Nature* **508,** 113–117 (2014).

13. Wu, M., Pastor-Pareja, J. C. & Xu, T. Interaction between RasV12 and scribbled clones induces tumour growth and invasion. *Nature* **463,** 545–548 (2010).

14. Archetti, M., Ferraro, D. A. & Christofori, G. Heterogeneity for IGF-II production maintained by public goods dynamics in neuroendocrine pancreatic cancer. *Proc. Natl. Acad. Sci. U. S. A.* **112,** 1833–1838 (2015).

15. Melo, S. A. *et al.* Cancer Exosomes Perform Cell-Independent MicroRNA Biogenesis and Promote Tumorigenesis. *Cancer Cell* **26,** 707–721 (2014).

16. Alfarouk, K. O., Muddathir, A. K. & Shayoub, M. E. A. Tumor acidity as evolutionary spite. *Cancers (Basel).* **3,** 408–414 (2011).

17. Carmona-Fontaine, C. *et al.* Emergence of spatial structure in the tumor microenvironment due to the Warburg effect. *Proc. Natl. Acad. Sci. U. S. A.* **110,** 19402–19407 (2013).

18. Montcourrier, P., Silver, I., Farnoud, R., Bird, I. & Rochefort, H. Breast cancer cells have a high capacity to acidify extracellular milieu by a dual mechanism. *Clin. Exp. Metastasis* **15,** 382–392 (1997).

19. Ghosh, S., Elankumaran, S. & Puri, I. K. Mathematical model of the role of intercellular signalling in intercellular cooperation during tumorigenesis. *Cell Prolif.* **44,** 192–203 (2011).

20. Durrett, R., Foo, J., Leder, K., Mayberry, J. & Michor, F. Evolutionary dynamics of tumor progression with random fitness values. *Theor. Popul. Biol.* **78,** 54–66 (2010).

21. Iwasa, Y. & Michor, F. Evolutionary Dynamics of Intratumor Heterogeneity. *PLoS One* **6,** e17866 (2011).

22. Durrett, R., Foo, J., Leder, K., Mayberry, J. & Michor, F. Intratumor Heterogeneity in Evolutionary Models of Tumor Progression. *Genetics* **188,** 461–477 (2011).

23. Roche, B., Drake, J. M. & Rohani, P. An agent-based model to study the epidemiological and evolutionary dynamics of Influenza viruses. *BMC Bioinformatics* **12,** 87 (2011).
